# Adverse drug reaction assessment of pembrolizumab and nivolumab in esophageal cancer treatment based on the US FAERS database

**DOI:** 10.3389/fimmu.2026.1738351

**Published:** 2026-04-02

**Authors:** Jinhan Chen, Qian Xu, Yuou Ying, Jinsheng Yu, Yi Liu, Fangmin Zhao, Qijin Shu

**Affiliations:** 1 The First School of Clinical Medical, Zhejiang Chinese Medical University, Hangzhou, China; 2 Department of Oncology, The First Affiliated Hospital of Zhejiang Chinese Medical University (Zhejiang Provincial Hospital of Chinese Medicine), Hangzhou, China

**Keywords:** adverse events, esophageal cancer, immunotherapy, nivolumab, pembrolizumab

## Abstract

**Background:**

Esophageal cancer (EC) is one of the most aggressive malignant tumors, associated with high mortality rates. Pembrolizumab and nivolumab have been incorporated into immunotherapy regimens for advanced EC, but immune-related adverse events (irAEs) remain a major factor affecting antitumor efficacy. This study aims to conduct a comprehensive and systematic analysis of adverse events (AEs) associated with pembrolizumab and nivolumab in EC immunotherapy using the FAERS database, providing new insights to optimize clinical practice.

**Methods:**

All AE reports related to pembrolizumab in EC were extracted from the FAERS database. Disproportionality analyses were performed using three algorithms, including the ROR, PRR, BCPNN. Based on Kaplan-Meier analysis with log-rank tests, the median time to onset and associated risk factors for relevant AEs were determined. Finally, univariable logistic regression analysis was used to identify risk factors for death associated with AEs.

**Result:**

A total of 3,669 AE reports related to pembrolizumab and 4,308 AE reports related to nivolumab were identified. Descriptive analysis showed that the number of AE reports increased year by year, and the number of reports was higher in the elderly population. The United States, Japan, and China were the primary reporting countries. The overall median time to onset for pembrolizumab-related PTs was 35 days, while that for nivolumab-related PTs was 52 days. In pembrolizumab, age ≥ 65 years was a risk factor for shorter median onset time, whereas in nivolumab, female gender was a risk factor for shorter median onset time. Among pembrolizumab-related AEs, weight was associated with a higher mortality risk. For nivolumab-related AEs, age, weight, increased cumulative number of adverse reactions, and earlier median onset time were associated with higher mortality risk.

**Conclusion:**

This real-world study is the first to analyze pembrolizumab and nivolumab irAEs in EC using the FAERS database, identifying high-signal events such as endocrine, hepatic, renal, gastrointestinal toxicities. Additional cardiovascular and pulmonary events are observed. Female and older patients experienced earlier irAEs. Increased mortality risk was associated with weight, cumulative number of AEs, and earlier irAE onset. These findings can guide personalized treatment, early intervention, and improve prognosis.

## 1 Introduction

Global cancer statistics data showed that in 2022, there were approximately 510,716 new cases of esophageal cancer (EC) (accounting for 2.6% of all sites) and 445,129 deaths (representing 4.6% of all sites) (1). EC was a relatively common digestive system tumor characterized by high invasiveness, often detected at an advanced stage. Traditional treatment methods include surgery, radiotherapy, and chemotherapy, which have limited clinical efficacy and significant toxic side effects, seriously affecting the survival prognosis of patients (2–4). In recent years, immune checkpoint inhibitors represented by PD-1 inhibitors have brought new opportunities for the treatment of EC (5, 6). Studies have revealed that PD-1 inhibitors demonstrate anti-tumor activity by blocking the PD-1 and PD-L1 pathway and reactivating T cell immune function (7, 8). Based on the KEYNOTE-590 and ATTRACTION-3 trials, pembrolizumab and nivolumab have become one of the standard treatment regimens for advanced EC (9, 10). However, while immune checkpoint inhibitors (ICIs) such as pembrolizumab and nivolumab reactivate T cells to exert anti-tumor effects, they may also cause excessive activation of the immune system, thereby triggering a series of unique immune-related adverse events (irAEs) (11). These irAEs involve multiple organ systems, including the skin, gastrointestinal tract, liver, and thyroid gland, which may even threaten life in severe cases (12, 13). Although randomized controlled trials (RCTs) have confirmed the efficacy and relatively controllable safety of pembrolizumab and nivolumab in EC, the strict inclusion criteria and limited sample sizes of RCTs often make it difficult to fully and accurately reveal the full-spectrum adverse reaction characteristics of these drugs in real-world clinical practice, especially the risks in rare, delayed-onset, or high-risk populations. The FDA Adverse Event Reporting System (FAERS) database served as the repository for adverse events (AEs) on drugs and biological products regulated by the FDA. It collected numerous AEs in clinical practice and enabled early identification of safety concerns, guided regulatory interventions, and supported evidence-based clinical decision-making (14–16). Therefore, this study utilizes the FAERS database to conduct comprehensive data mining and comparative analysis on the adverse reactions of pembrolizumab and nivolumab in the treatment of EC. The purpose is to reveal their real-world safety profiles, identify differential risk signals, and provide key evidence-based support for clinical medication decision-making and risk management.

## 2 Methods

### 2.1 Data sources

This study conducted a pharmacovigilance analysis of adverse reactions associated with pembrolizumab and nivolumab in EC immunotherapy using data from the FAERS database. The FAERS database comprises seven datasets: demographic and management information (DEMO), drug information (DRUG), adverse event information (REAC), patient outcome information (OUTC), report source information (RPSR), Therapy drug therapy start and end dates (THER), and indication/diagnosis information (INDI) (17, 18).

### 2.2 Data deduplication and extraction

This study used publicly available FAERS data from 2004 Q1 to 2025 Q2 (https://fis.fda.gov/extensions/FPD-QDE-FAERS/FPD-QDE-FAERS.html). Data were imported the ASCII data (2004 Q1 to 2025 Q2) package downloaded from the FAERS database into R statistical analysis software version 4.4.2 for data integration, cleaning, and standardization. Potential drug names were collected from sources like FDA Website (https://www.accessdata.fda.gov/scripts/cder/daf/index.cfm), and PubMed Medical subject headings. The active ingredients, generic names and trade names of the two medicines have been determined as follows. (1) pembrolizumab: “pembrolizumab”, “keytruda”, “mk-3475”; (2) nivolumab: “nivolumab”, “opdivo”, “opdyta”, “bms-936558”. The DRUG table’s “drugname” and “prod_ai” fields were filtered, with the “role_cod” (drug role code) field restricted to “PS” (primary suspect) in the DRUG table while restricting “indi_pt” to EC. The “drugname” and “indi_pt” of pembrolizumab and nivolumab in the DRUG and INDI table was listed in **Supplementary Table 1**. AE reports were coded using the preferred terms (PT) from the Medical Dictionary for Drug Regulatory Activities (MedDRA) version 27.1 and classified according to the System Organ Class (SOC). Following the FDA-recommended method for removing duplicate reports, the PRIMARYID, CASEID, and FDA_DT fields were selected from the DEMO table. The data was sorted by CASEID, FDA_DT, and PRIMARYID. For reports with the same CASEID, the report with the highest FDA_DT value was retained, as a higher value indicates a newer report date. For reports with identical CASEID and FDA_DT values, the report with the largest PRIMARYID value was kept (19). To ensure the completeness of subsequent descriptive analyses and the calculation of ae risk signals, the reporting of missing values for variables such as gender, age, and weight in the data was retained.

### 2.3 Statistical methods

**Figure 1:**
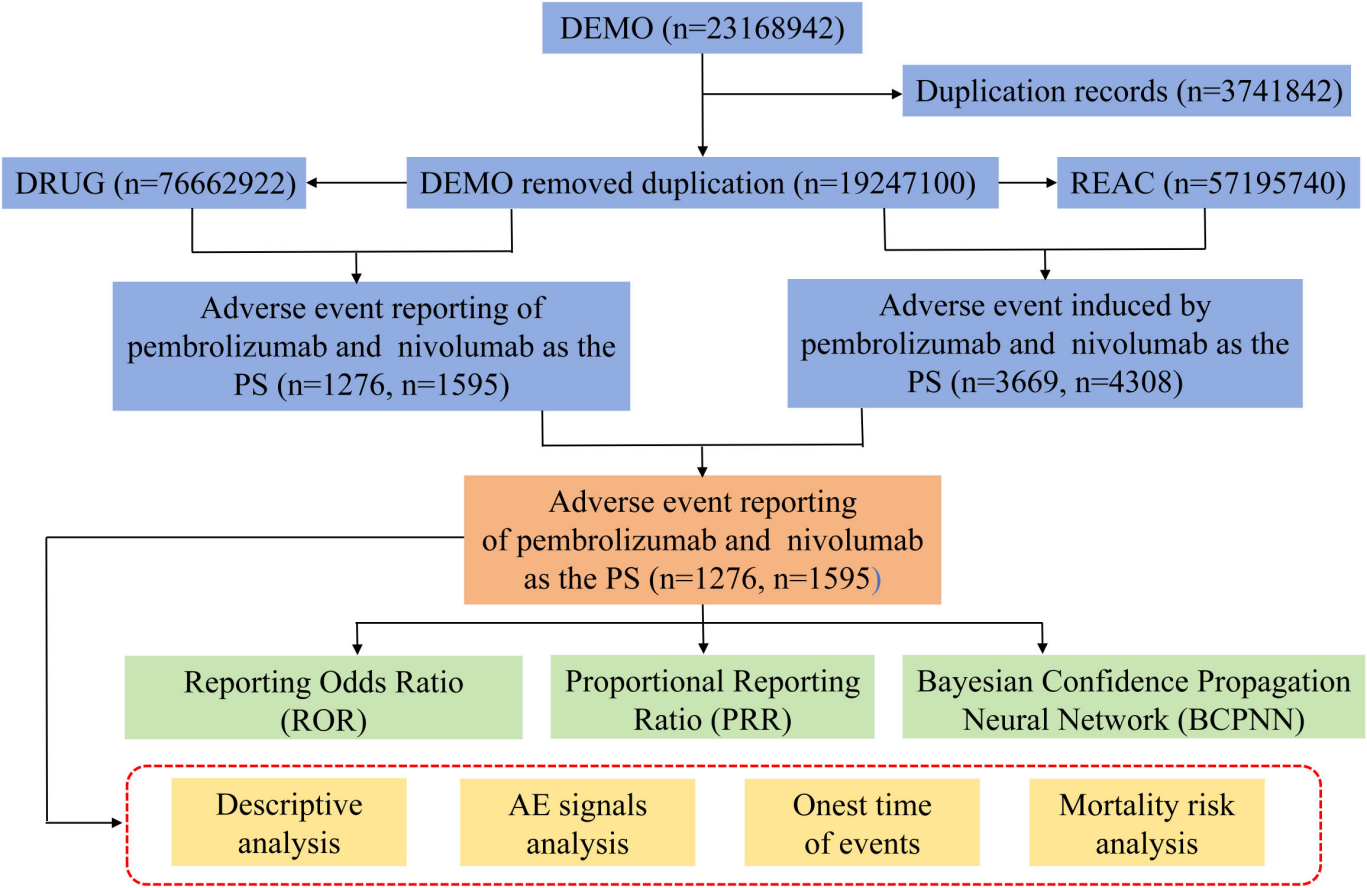
FAERS data extraction and study flow chart.

In this study, R 4.4.2 and SPSS 29.0 was used for statistical analysis. Descriptive analyses were performed to characterize the clinical profiles of esophageal cancer patients experiencing pembrolizumab and nivolumab-related AEs in the FAERS database. Categorical variables for all reports were presented as numbers and percentages. Three distinct methodologies were utilized to assess signal strength and the potential risk of AEs: the reporting odds ratio (ROR), proportional reporting ratio (PRR), Bayesian confidence propagation neural network (BCPNN). Quantitative comparisons of AE risks among two drugs were conducted using the values of ROR, PRR, information component (IC) (**Table 1**). In order to conduct subsequent analysis on time to onset of AEs and risk factors related to death outcomes, reported cases with missing values of gender, age, and weight in the DEMO table were further deleted. All missing values were integrated by deletion independently based on individual factors. The time to onset of AEs was assessed by calculating the duration between the initiation date of the primary suspected drug and the date of the first reported AEs. The median time to onset AEs associated with pembrolizumab and nivolumab analyzed by Kaplan–Meier analysis with log-rank tests. And the time to onset in Subgroup of age, gender, weight, each SOC, and each irAE were also analyzed by Kaplan-Meier analysis with log-rank tests. The associations of gender, age, weight, the number of AEs and the time of the first occurrence of AEs with the increased risk of mortality outcomes were compared by univariable logistic regression. The specific research process of this study is shown in **Figure 1**.

**Table 1:** Overview of algorithms utilized for signal detection.

Algorithms	Formulas	Criteria
ROR	$\text{ROR = }\frac{\left(a/c\right)}{\left(b/d\right)}=\frac{ad}{bc}$ ${95\%CI = e}^{\ln(\text{ROR})\pm 1.96}\sqrt{\left(\frac{1}{\text{a}}+\frac{1}{\text{b}}+\frac{1}{\text{c}}+\frac{1}{\text{d}}\right)}$	a ≥ 3, The lower limit of 95% CI (RORL) > 1
PRR	$\text{PRR}=\frac{a/(a+b)}{c/(c+d)}$ ${95\%CI=e}^{\ln(\text{PRR)}\pm 1.96}\sqrt{\frac{1}{a}-\frac{1}{a+b}+\frac{1}{c}-\frac{1}{\text{c+d}}}$	a ≥ 3, PRR ≥ 2, χ² ≥ 4
	${\text{χ}}^{2}=\frac{{(\text{ad - bc)}}^{2}(\text{a + b + c + d)}}{(\text{a + b)(a + c)(c + d)(b + d)}}$	
BCPNN	$\alpha_{i}=\beta_{j}\;=\;1,\alpha =\beta \;=\;2,\gamma_{ij}\;=\;1$ N=a+b+c+d	a ≥ 3, The lower limit of 95% CI (IC025) > 0
	${\text{IC = log}}_{2}\frac{a(a+b+c+d)}{\left(a+b)(a+c\right)}$ ${\text{E(IC) = log}}_{2}\frac{\left(a+\gamma_{ij})(N+a)(N+\beta \right)}{\left(N+\gamma )(a+b+a_{i})(a+c+\beta_{j}\right)}$ $\text{V(IC)=}{\left(\frac{1}{{\text{ln}}_{2}}\right)}^{2}[\frac{N-a+\gamma -\gamma_{ij}}{\left(a+\gamma_{ij})(1+N+\gamma \right)}+\frac{N-a-b+a-a_{i}}{\left(a+b+a_{i})(l+N+a\right)}+\frac{N-a-c+\beta -\beta_{j}}{\left(a+c+\beta_{j})(l+N+\beta \right)}]$ $\text{SD = }\sqrt{\text{V(IC)}}$ IC025=E(IC)-2SD	


a, number of reports containing both the target drug and target adverse drug reaction; b, number of reports containing other adverse drug reaction of the target drug; c, number of reports containing the target adverse drug reaction of other drugs; d, umber of reports containing other drugs and other adverse drug reactions. 95%CI, 95% confidence interval; χ², Chi-squared; IC, information component; IC025, the lower of 95%CI of the IC.

## 3 Results

### 3.1 Descriptive analysis

**Figure 2:**
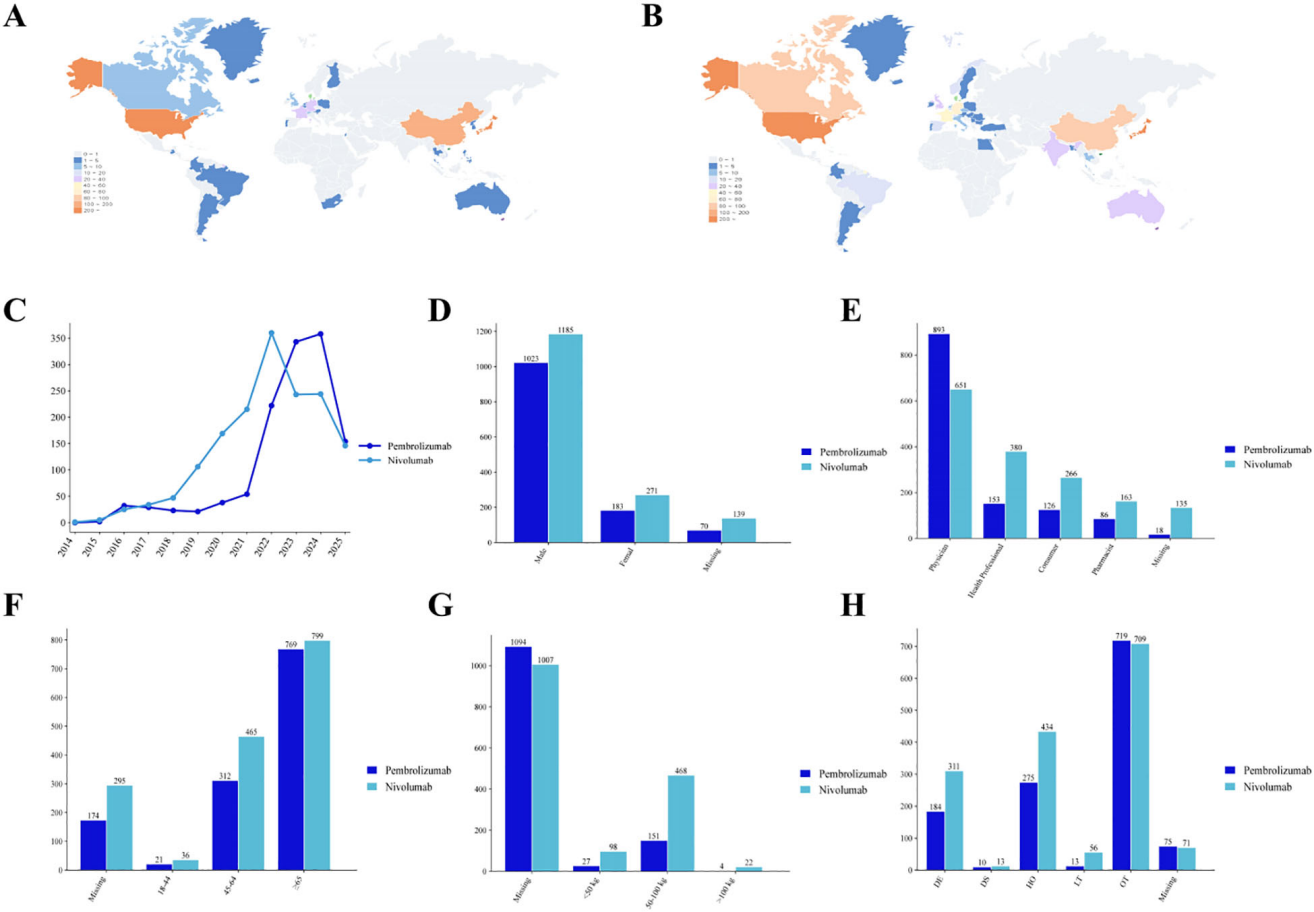
Distribution of demographic characteristics of patients with AEs associated with pembrolizumab and nivolumab treatment in EC. **(A)** Distribution of countries in pembrolizumab; **(B)** countries in nivolumab; **(C)** Distribution of reporting years; **(D)** Distribution of gender; **(F)** Distribution of age; **(G)** Distribution of weight; **(H)** Distribution of outcomes.

From the Q1–2004 to Q2 2025, a total of 3,669 AE reports related to pembrolizumab and 4,308 reports related to nivolumab for EC treatment were retrieved from the FAERS database. These reports involved 1,276 patients treated with pembrolizumab and 1,595 patients treated with nivolumab (**Figure 1**) (**Supplementary Table 2**). In the national distribution, Japan (61.7% and 38.6%), the United States (18.3% and 28.3%), and China (9.4% and 5.9%) reported the highest numbers (**Figures 2A, B**). AEs associated with the immunotherapy of EC using pembrolizumab and nivolumab have been reported since 2014. Regarding the annual distribution of AE reports, both pembrolizumab and nivolumab showed year-on-year increases in reported cases. Pembrolizumab experienced a peak growth period from 2022 to 2024, while nivolumab exhibited a peak growth period from 2021 to 2024 (**Figure 2C**). About gender differences, AE reports from males accounted for 80.2% and 74.3%, significantly higher than those from females at 14.3% and 17.0% (**Figure 2D**). Physicians submitted the highest number of reports (70.0% and 40.8%), followed by healthcare professionals (12.0% and 23.8%) and consumers (9.9% and 16.7%) (**Figure 2E**). By age, the highest number of reports was reported in those aged 65 years and older (60.3% and 50.1%), followed by those aged 45 to 64 years (24.4% and 29.2%) and those aged 18 to 44 years (1.6% and 2.3%) (**Figure 2F**). In terms of weight distribution, the 50–100 kg range was the most common (11.8% and 29.3%) (**Figure 2G**). The hospitalization rate and mortality rate for patients with pembrolizumab-related AEs was 21.6% and 14.4%. For patients with nivolumab-related AEs, the hospitalization rate was 27.2% and the mortality rate was 19.5% (**Figure 2H**).

### 3.2 Frequency and intensity distribution of AE signals

**Figure 3:**
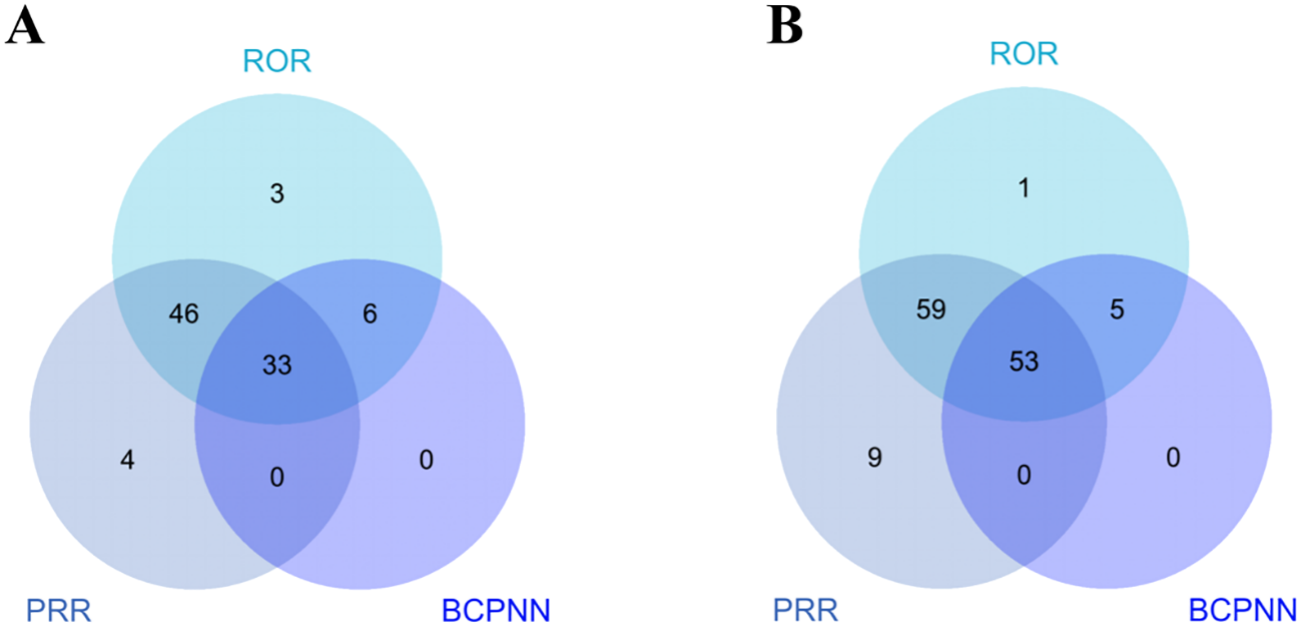
Venn diagram of PT signal detection associated with pembrolizumab and nivolumab treatment for EC. **(A)** pembrolizumab; **(B)** nivolumab.

**Figure 4:**
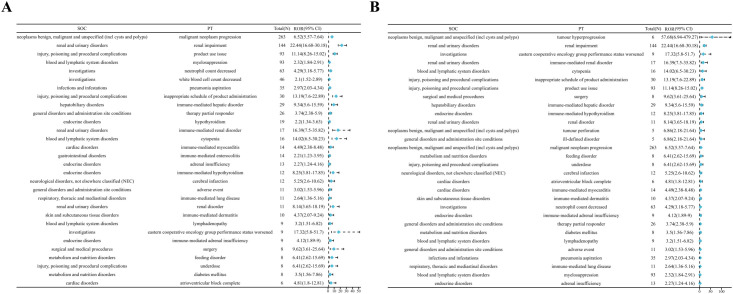
**(A)** Top 30 signal frequencies of AEs at the SOC level for pembrolizumab in the treatment of EC. **(B)** Signal strength of AEs at the SOC level for pembrolizumab in the treatment of EC.

Thirty-three positive PTs were found in pembrolizumab and fifty-three positive PTs in nivolumab (**Figure 3**) (**Supplementary Tables 3a, b**). Among AEs associated with pembrolizumab, malignant neoplasm progression and renal impairment were the most common. Adverse reactions observed in renal and urinary disorders included renal impairment and immune-mediated renal disorder. Blood and lymphatic system disorders comprised myelosuppression and lymphadenopathy. Hepatobiliary disorders include immune-mediated hepatic disorder. Endocrine disorders include hypothyroidism or immune-mediated, adrenal insufficiency or immune-mediated. Cardiac disorders include immune-mediated myocarditis, atrioventricular block complete. Gastrointestinal disorders include immune-mediated enterocolitis. Respiratory, thoracic and mediastinal disorders include immune-mediated lung disease. Skin and subcutaneous tissue disorders include immune-mediated dermatitis. Neurological disorders, not elsewhere classified (NEC) include cerebral infarction. Other AEs include neutrophil count decreased, white blood cell count decreased, and pneumonia aspiration etc. (**Figure 4A**). Among AEs with positive signals associated with pembrolizumab use, the top 30 AEs ranked by signal strength are shown in the. Immune-related AEs such as renal impairment or immune-mediated renal disorder, immune-mediated hepatic disorder, immune-mediated hypothyroidism, immune-mediated myocarditis, and immune-mediated dermatitis carry a high risk signal intensity (**Figure 4B**).

**Figure 5:**
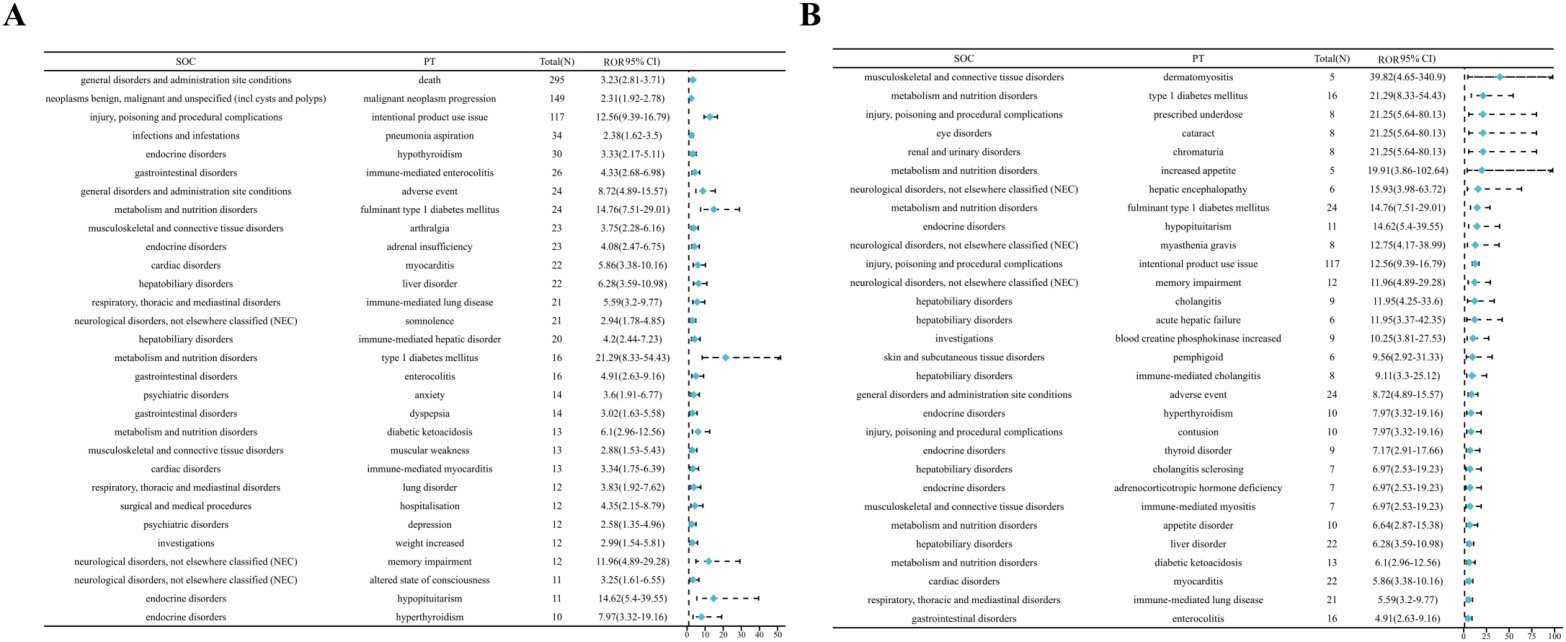
**(A)** Top 30 signal frequencies of AEs at the SOC level for nivolumab in the treatment of EC. **(B)** Signal strength of AEs at the SOC level for nivolumab in the treatment of EC.

**Figure 6:**
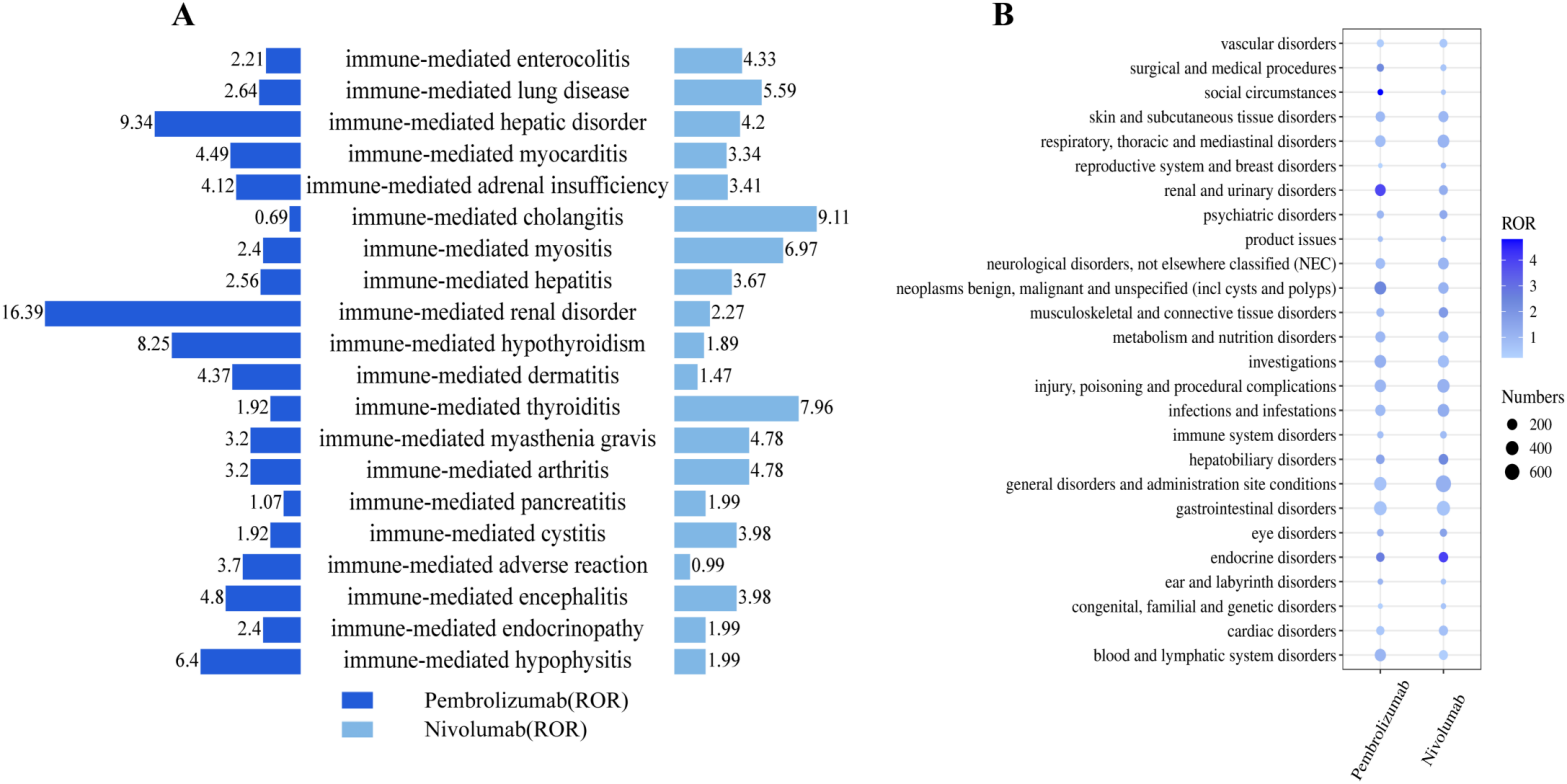
**(A)** Pembrolizumab and nivolumab ROR ratio of immune-induced AEs in the treatment of EC. **(B)** Bubble chart showing the frequency and intensity distribution of SOC in immunotherapy with pembrolizumab and nivolumab for EC.

Among AEs associated with nivolumab treatment, death and malignant neoplasm progression were the most common. Endocrine system disorders included hypothyroidism, hyperthyroidism, adrenal insufficiency, and hypopituitarism. Gastrointestinal system disorders comprised immune-mediated enterocolitis and dyspepsia. Hepatobiliary disorders included immune-mediated hepatic disorder. Neurological disorders, not elsewhere classified (NEC) included somnolence and altered mental status. Cardiovascular disorders included myocarditis. Respiratory, thoracic and mediastinal disorders included immune-mediated lung disease. Other related AEs observed included diabetes mellitus, anxiety/depression, and pneumonia aspiration etc. (**Figure 5A**). Among the positive signal AEs associated with the use of nivolumab, the top 30 PTs were ranked according to signal intensity. The signal intensity of dermatomyositis was the highest. Subsequently, high-risk adverse reactions observed within the hepatobiliary system category include hepatic encephalopathy, cholangitis, acute liver failure, and immune-mediated cholangitis (**Figure 5B**). Finally, visual analysis showed ROR differences in irAEs associated with pembrolizumab versus nivolumab (**Figure 6A**).

### 3.3 SOC signal detection

**Figure 7:**
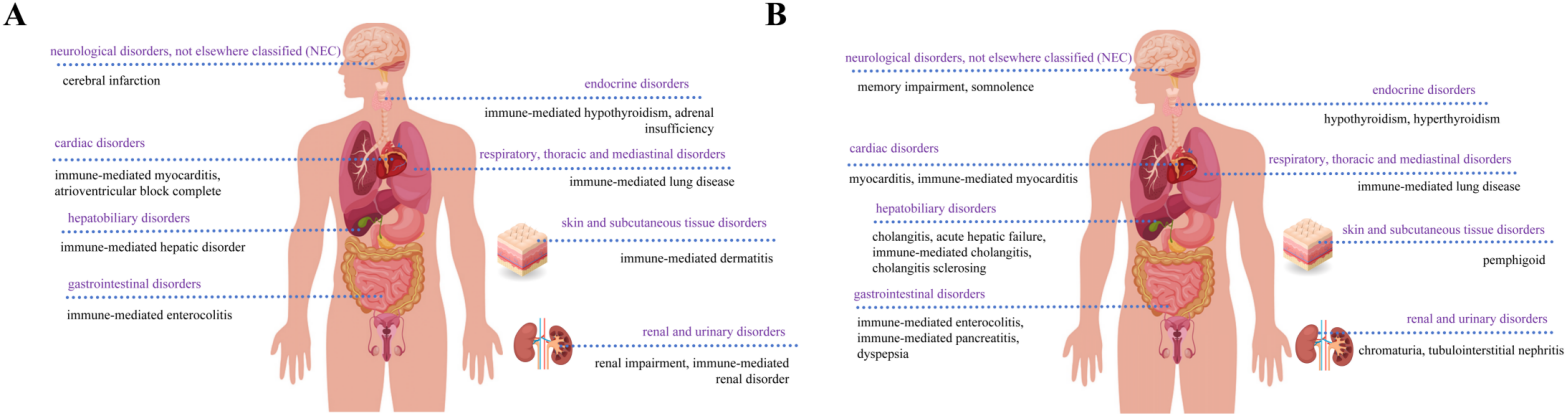
**(A)** Graphical summary of organ distribution of AEs associated with pembrolizumab therapy in EC. **(B)** Graphical summary of organ distribution of AEs associated with nivolumab therapy in EC.

Pembrolizumab and nivolumab-related AEs occurred across 27 SOC in the FAERS database, indicating that PD-1-related AEs commonly affect multiple organs (**Supplementary Tables 3c, d**). Endocrine disorders and renal and urinary disorders met all three algorithms simultaneously in both drugs. Gastrointestinal disorders, general disorders and administration site conditions were the most frequent SOCs for both drugs. For pembrolizumab, hematologic and lymphatic disorders and renal and urinary disorders were reported in the highest numbers. For nivolumab, there were higher numbers of reports of respiratory disorders and hepatobiliary disorders. In the risk-signal intensity ranking, pembrolizumab showed higher signals for renal and urinary disorders, whereas nivolumab showed higher signals for hepatobiliary disorders. The distribution and severity of each SOC are presented in a bubble plot (**Figure 6B**). In addition, graphical summaries were used to summarize the differential distribution of the two drugs in each major SOC (**Figure 7**).

### 3.4 Subgroup analysis by gender and age

Considering gender and age differences in drug safety assessments may aid in the precise management of AEs. Therefore, we further explored the distribution of positive signals for AEs associated with pembrolizumab and nivolumab treatment of EC after stratification by gender and age subgroups (**Supplementary Tables 4a-h**). Results showed that males experienced more AEs than females for both drugs. For pembrolizumab, both males and females experienced renal impairment, product use issues, myelosuppression, and neutrophil count decreased. For nivolumab, both males and females experienced death, intentional product use issue, myocarditis, and liver disorder. In the male cohorts of both drugs, pneumonia aspiration, immune-mediated hepatic disorder, hypothyroidism, immune-mediated myocarditis, and immune-mediated lung disease occurred. No common AEs were identified in the female cohorts of both drugs. We conducted a subgroup analysis based on age differences in the enrolled population, dividing participants into two groups: ≥65 years and <65 years. In pembrolizumab, the ≥65-year-old elderly population identified more AEs, including decreased white blood cell or blood cell counts, infection with aspiration pneumonia, hyponatremia, and esophageal stricture. In nivolumab, the ≥65 age group similarly identified more relevant AEs, including myasthenia gravis, hypopituitarism, adrenal insufficiency, arthralgia, and sclerosing cholangitis. Notably, In both pembrolizumab and nivolumab, the elderly population was found to be more susceptible to neurological disorders, including cerebral infarction, anxiety/depression, memory impairment, or altered mental status.

### 3.5 Analysis of time to onset and influencing factors

**Figure 8:**
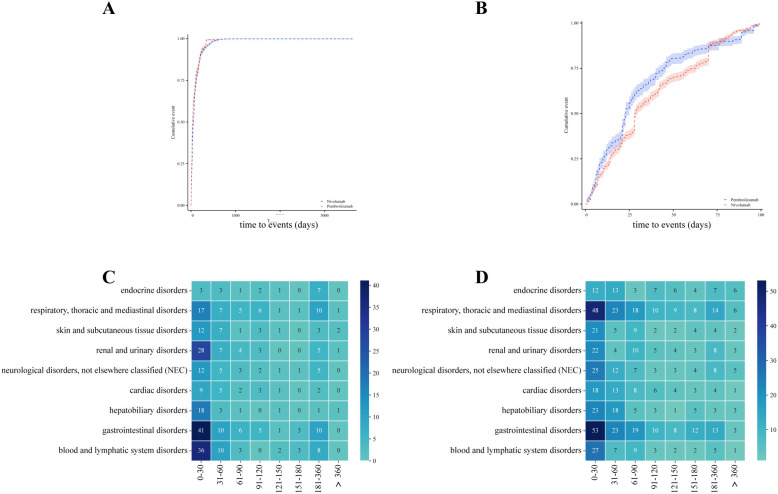
**(A)** Cumulative probability curve for median time to onset of AEs associated with pembrolizumab therapy in EC. **(B)** Cumulative probability curve for median time to onset at the first 100 days of AEs associated with pembrolizumab therapy in EC. **(C)** Heatmap of timing distribution of major SOC associated with pembrolizumab therapy in EC. **(D)** Heatmap of timing distribution of major SOC associated with nivolumab therapy in EC.

The overall median time to onset for pembrolizumab-related PTs was 35 days (95%CI: 29–40 days), while that for nivolumab-related PTs was 52 days (95%CI: 46–58 days). During the first 100 days, the cumulative rate of pembrolizumab-related PTs was faster than that of nivolumab-related PTs. In an exploratory analysis of median onset times for PTs in organs of interest SOC, both pembrolizumab and nivolumab related PTs showed median onset times concentrated within the first 3 months (**Figure 8**). In pembrolizumab, the median onset times for renal and urinary disorders, hepatobiliary disorders, and gastrointestinal disorders were relatively early. In nivolumab, hepatobiliary disorders had the earliest median onset time, while renal and urinary disorders had the latest. Endocrine disorders had the latest onset time in both groups (**Supplementary Table 5**). Finally, we identified factors influencing the median time to onset of the first esophageal cancer-related PT in both groups. In pembrolizumab, age of 65 years or older was a risk factor for shorter median onset time, whereas in nivolumab, female gender was a risk factor for shorter median onset time (**Table 2**).

**Table 2:** Risk factors for early time to onset of AEs associated with pembrolizumab and nivolumab in the treatment of EC.

Variables	Pembrolizumab		Nivolumab	
	Mean TTO (days) (95%CI)	*P*-value	Mean TTO (days) (95%CI)	*P*-value
Age (years)				
≥65	23 (21-33)	0.014*	54 (43-65)	0.88
<65	45 (27-76)		56 (44-67)	
Gender				
Male	27 (23-40)	0.158	58 (50-68)	0.006*
Female	28 (20-64)		43 (31-58)	
Weight (kg)				
>100	21 (5-39)	0.372	117 (24-147)	0.743
50-100	31 (23-46)		60 (50-71)	
<50	25 (8-39)		57 (42-72)	

Mean TTO, median time to onset; 95%CI, 95% confidence interval; *p < 0.05.

### 3.6 Factors influencing mortality risk in the AE population

Univariable logistic regression analysis was used to identify mortality risk factors for AEs induced by pembrolizumab and nivolumab. Among pembrolizumab-related AEs, weight exceeding 100 kg was associated with a higher mortality risk. For nivolumab-related AEs, age of 65 years or older, weight less than 50 kg, cumulative AEs, and time to first AE (days) were associated with higher mortality risk (**Table 3**).

**Table 3:** Risk factors for death associated with pembrolizumab and nivolumab in the treatment of EC.

Variables	Pembrolizumab		Nivolumab	
	OR (95%CI)	*P*-value	OR (95%CI)	*P*-value
Age (years)				
≥65	0.948 (0.266-3.17)	0.892	7.041 (1.78-29.546)	0.008*
44-64	1.579 (0.451-5.525)	0.475	2.567 (0.601-10.957)	0.203
18-44	Reference (OR = 1.00)		Reference (OR = 1.00)	
Gender				
Male	Reference (OR = 1.00)		Reference (OR = 1.00)	
Female	1.304 (0.857-1.986)	0.216	0.989 (0.714-1.370)	0.948
weight (kg)				
>100	8.347 (1.111-64.074)	0.039*	0.588 (0.213-1.624)	0.306
50-100	Reference (OR = 1.00)		Reference (OR = 1.00)	
<50	0.675 (0.146-3.119)	0.615	1.564 (1.004-2.435)	0.048*
Cumulative AEs	0.969 (0.910-1.031)	0.318	1.03 (1.003-1.058)	0.030*
Time to first AE (days)	1.002 (0.998-1.005)	0.384	0.972 (0.967-0.977)	0.000*

OR, Odds Ratio; 95%CI, 95% confidence interval; *p < 0.05.

## 4 Discussion

### 4.1 Basic characteristics of AEs reported cases in immunotherapy for EC with pembrolizumab and nivolumab

In observations of demographic characteristics, we found that AEs associated with pembrolizumab and nivolumab immunotherapy for EC primarily occurred in males. Previous studies indicate that the age-standardized incidence ratio (ASIR) for esophageal adenocarcinoma (EAC) is 6.7:1 and for esophageal squamous cell carcinoma (ESCC) is 3.3:1 (20). The male predominance in incidence across different histopathologies may be one factor contributing to the concentration of immune-related treatment-related adverse events in males. Both PD-1 irAEs predominantly occurred in elderly patients aged over 65. Global EC incidence patterns show a gradual increase with age, peaking between 85 and 89 years, indicating a high-risk association with diagnosis in the elderly (21). Clinicians should pay particular attention to the occurrence of related AEs in elderly males. Analysis of reporters revealed that the majority were healthcare professionals, suggesting strong reliability of the reported AEs in the database. The United States, Japan, and China reported the highest number of AEs, reflecting regional variations in attention to irAEs for EC. However, this may be related to the difference in the size of the drug population in each country.

### 4.2 Distribution and correlation of AEs in immunotherapy for EC with pembrolizumab and nivolumab

The findings indicate that immune-mediated endocrine disorders are particularly prominent in immunotherapy for EC. Concurrently, immune-mediated hepatic and renal injury also exert significant impact. Furthermore, a broad spectrum of immune-mediated autoimmune reactions including small bowel colitis, cholangitis, dermatitis, pneumonia, and myocarditis affecting multiple organs has been observed. In order to further compare the similarities and differences in the adverse reactions between pembrolizumab and nivolumab in the treatment of EC as shown in the research results and clinical trials, we additionally reviewed the data results of 5 clinical trials including KEYNOTE-590 (9), KEYNOTE-180 (22), KEYNOTE-181 (23), IRB no. 2019-01-147 (24), and ATTRACTION-3 (10) (**Supplementary Table 6**). The clinical trial results also showed immune damage in multiple organs and systems. Particularly in the KEYNOTE-590 clinical trial, after adding pembrolizumab treatment, it was shown to have a higher incidence of endocrine system disorders compared to other adverse reactions, including 40 cases (11%) of hypothyroidism, 21 cases (6%) of hyperthyroidism, and rare endocrine events such as adrenal insufficiency, pituitary inflammation, and type 1 diabetes also occurred in one case each. For liver and kidney injury events, the number of occurrences in the clinical trial results was not many. In KEYNOTE-181, there were 6 cases (1.9%) of hepatitis and 1 case (< 1%) of nephritis in KEYNOTE-590. Based on the large sample support from the FAERS database, extensive liver and kidney injuries were magnified. Other notable events include pneumonia, myocarditis, pancreatitis, etc., similar to the FAERS database, the reported cases were relatively rare, but they posed significant challenges to the efficacy and prognosis of patients’ anti-tumor treatment. Therefore, elucidating the underlying mechanisms and strengthening early management are crucial for enhancing clinical treatment safety.

IrAEs such as thyroiditis, hypophysitis, adrenal insufficiency, and insulin-deficient diabetes constitute endocrine toxicity in ICI therapy for EC. These events fundamentally result from loss of immune tolerance and subsequent immune attacks targeting endocrine organs (25, 26). Studies indicate that patients experiencing endocrine irAEs demonstrate significantly prolonged overall survival (OS) and progression-free survival (PFS) compared to those without irAEs (27), suggesting that alterations in the immune microenvironment of endocrine organs may reflect the intensity of systemic antitumor immune responses. Research indicates that thyroid dysfunction is prevalent in anti-PD-1 therapy (28). Potential mechanisms may include abnormal activation of self-reactive CD8^+^ T cells and thyroid infiltration induced by disruption of peripheral immune tolerance (29). Additionally, increased cytokine production and inflammatory responses further damage endocrine tissues, such as IFN-γ and TNF-α-mediated thyroid follicular cell apoptosis as well as elevated thyroid peroxidase antibodies (TPOAb). Related studies have identified elevated thyroid stimulating hormone (TSH), positive anti-thyroglobulin antibodies (TGAb), and positive TPOAb as risk factors for endocrine irAEs, validated from a genetic perspective (30), highlighting the importance of dynamic monitoring of these indicators for risk stratification in clinical management. Consistent with previous reports, hypophysitis was infrequently reported in patients receiving anti-PD-1 therapy, while cases of insulin-deficient diabetes and adrenal insufficiency remained rare.

In this study, pembrolizumab was more strongly associated with nephrotoxicity than nivolumab during immunotherapy for EC. Previous studies have also confirmed the findings of this research. Zhu J and colleagues concluded through meta-analysis and FAERS database analysis that the risk of acute kidney injury associated with cancer immunotherapy was higher for pembrolizumab (RR = 1.60; 95% CI: 1.17–2.20) was higher than that of nivolumab (RR = 1.23; 95% CI: 0.78–1.93). AE signal detection also observed a higher signal intensity for pembrolizumab (ROR = 2.27) compared to nivolumab (ROR = 2.05) (31). Immune-mediated nephrotoxicity arises from impaired immune tolerance following loss of T-cell suppression. Reports indicate that ICI-induced acute kidney injury (AKI) correlates with higher clinical mortality (32). Key complications include volume overload, electrolyte disturbances, arrhythmias, and diabetes insipidus. Acute interstitial nephritis (AIN) is the most common pathological finding in ICI-associated AKI, characterized by varying degrees of infiltration by CD4^+^ T lymphocytes, plasma cells, and eosinophils (33). Studies indicate that the use of other nephrotoxic medications (such as proton pump inhibitors and NSAIDs) and pre-existing chronic kidney disease increase susceptibility to immune-mediated renal injury during PD-1 inhibitor therapy (34, 35).

In the SOC comparison, the reported signal intensity of liver disease was observed to be similar for both drugs. A pharmacovigilance study by Xu Z et al. found similar overall hepatotoxicity between the two drugs (pembrolizumab ROR = 1.57; nivolumab ROR = 1.44) (36). Immune checkpoint inhibitors enhance immunity by blocking the PD-1 pathway and eliminating T-cell inhibitory signals. This not only targets tumor cells but also attacks normal hepatocytes and cholangiocytes, leading to hepatitis or cholangitis. Affected tissues often exhibit hepatic lobular inflammation, bile duct injury (37), and CD8^+^ T-cell infiltration, or granulomatous hepatitis and cholestatic injury (38). Severe cases may involve cytokine storms and steep rises in inflammatory mediators, further amplifying immune-mediated injury (39). However, unlike previous studies, we found that nivolumab carries a stronger positive signal of immune-mediated hepatitis and cholangitis than pembrolizumab. In addition, two unique positive signals for acute hepatic failure and hepatic encephalopathy were identified with nivolumab. Cases of nivolumab-associated fulminant hepatitis have been reported previously, and therefore, clinicians need to exercise a degree of vigilance in this regard (40, 41).

SOC frequency analysis indicates gastrointestinal toxicity as the most common related AE shared by both drugs. Case reports and series studies suggest that small intestinal colitis induced by these medications typically manifests as diarrhea, abdominal pain, and elevated inflammatory markers. Current research suggests the pathogenesis of immune-mediated enterocolitis may be linked to antimicrobial immunity. Certain bacteria within the intestinal lumen can induce inflammatory bowel disease (IBD) associated pathogenic Th1/Th17 responses, while others are associated with the regulation of Tregs and regulatory B cells (42–44). Mucosa-associated innate T cells (MAIT cells) also highlight the importance of the microbiota (45). Secondly, alterations in bacterial strains may also constitute a potential pathogenesis pathway (46). IMC patients primarily exhibit epithelial lymphocyte and neutrophil infiltration in colonic tissue (47). Therefore, when colonoscopy results are normal, pathological examination should be performed when necessary for definitive diagnosis. For patients with severe colitis, corticosteroid therapy may be considered, and immunotherapy should be discontinued when indicated. In this study, immune-mediated enterocolitis was observed concurrently with pembrolizumab and nivolumab. In some patients following radical esophagectomy, the gastrointestinal dysfunctions induced by anatomical alterations such as gastric lifting, coupled with neurological injuries contributing to conditions like gastroparesis and intestinal dysregulation, were associated with an increased susceptibility to superimposed immune-mediated gastrointestinal AEs (48). Therefore, during immunotherapy, patients require close monitoring of abdominal signs and gastrointestinal symptoms such as abdominal pain, diarrhea, and decreased appetite to promptly intervene in gastrointestinal toxicity reactions associated with immunotherapy.

Additionally, immune-related pulmonary toxicity has been observed. Previous studies have confirmed the risk of pneumonia in patients treated with PD-1 inhibitors (49, 50). Immunomediated pneumonia may overlap with infectious pneumonia and diffuse alveolar damage due to its nonspecific diagnostic and radiological features (51). Checkpoint inhibitor pneumonitis (CIP) significantly increases the risk of mortality and requires high-dose glucocorticoid therapy for short-term relief (52). Immune-mediated pneumonitis causes pulmonary injury and reduced lung function, while also promoting the development of infectious aspiration pneumonia. Corticosteroid therapy and pulmonary inflammation may compromise long-term antitumor regimens, potentially requiring dose reduction or discontinuation. Early, intermittent pulmonary CT screening is necessary to mitigate severe pulmonary symptoms associated with treatment interruption.

Cardiomyopathy represents a common AEs for both drugs within the cardiovascular system. Studies indicate that 90% of ICI-associated myocarditis patients exhibit elevated troponin levels. Myocarditis presenting solely with elevated cardiac biomarkers may be asymptomatic. However, myocarditis can manifest abruptly, potentially leading to cardiogenic shock and death (53). In addition, a positive safety signal for complete atrioventricular block was identified in association with pembrolizumab. Additionally, a signal for myocarditis with concomitant myasthenia gravis was observed with nivolumab treatment. Previous cases have reported triple M syndrome associated with PD-1 inhibitors, and this AEs has serious consequences (54, 55). Our results support clinicians to pay attention to the occurrence of this dangerous and serious combined AEs, through early electrocardiogram and blood markers characteristics, with continuous monitoring, Comprehensive management of immune-related cardiac adverse events through multidisciplinary team.

Subgroup analyses stratified by age showed that more positive signals of neurological AEs were found in the older population. Previous studies have indicated that depression or reduced mobility may serve as potential risk factors for older adults with advanced cancer experiencing higher-grade irAEs (56). Therefore, for elderly patients, it is essential to prevent neurological AEs associated with esophageal cancer immunotherapy through timely and critical guidance or nursing support, coupled with more precise management strategies (57).

### 4.3 Time to onset of AEs in immunotherapy for EC with pembrolizumab and nivolumab

This study systematically summarized the time to onset of irAEs associated with pembrolizumab and nivolumab immunotherapy. Overall, AEs occurred within the first two months of immunotherapy. Gastrointestinal system AEs associated with both drugs occurred earlier, whereas endocrine-related AEs occurred later. A previous study demonstrated that endocrine-related AEs develop slowly, with a median onset time of 61 days between 30 and 365 days (mean 117 days) after initiating PD-1/PD-L1 inhibitor therapy (58, 59). This is consistent with the onset timing of endocrine-related AEs observed in this study of EC immunotherapy, and similar delayed onset has been reported in comparable studies (60). Although the median onset time observed in this study aligns with previous reports (61, 62), a distinct finding was the earlier median onset time of renal-related AEs with pembrolizumab in EC immunotherapy. This may suggest the need for some early intervention for associated nephrotoxicity.

Log-rank analysis indicated that age ≥65 years was a risk factor for shorter median onset time in pembrolizumab, while female gender was a risk factor in nivolumab. Aging increases the risk of immune system tolerance loss. First, dendritic cells in the elderly secrete more pro-inflammatory factors and promote autoimmunity (63). Second, aging patients more frequently exhibit reduced production of immature T cells following thymic atrophy (64, 65). Additionally, studies have found that age-related gut microbiota dysbiosis disrupts the tolerogenic function of dendritic cells (DCs), leading to decreased regulatory T cell (Treg) induction and increased proinflammatory signaling (66). Therefore, older adults may be more susceptible to earlier onset of immune checkpoint irAEs. Fang Q et al., reviewing 3,795 patients receiving ICI therapy, similarly identified female sex as a predictor for early irAE onset in a multivariable logistic regression analysis (67). This may be attributed to women’s stronger immune responses (68).

### 4.4 Univariable logistic regression analysis of mortality risk

Finally, through univariable logistic regression analysis, we observed that weight, cumulative number of AEs, and median time to onset were risk factors associated with mortality in patients experiencing irAEs during immunotherapy. With pembrolizumab, patients weighing less than 50kg had a relative increased risk of death, whereas with nivolumab, patients weighing more than 100kg had a relative increased risk of death. This finding suggests that clinicians may need to be somewhat vigilant about extreme weight in the management of patients starting immunotherapy. Extreme underweight or extreme overweight may indicate disturbed nutritional status, and studies have shown that both malnutrition and overnutrition impair immune cell metabolism, reduce lymphocyte counts, and disrupt the balance between proinflammatory and anti-inflammatory responses. This results in reduced immune tolerance and increased susceptibility to infection or autoimmune responses (69, 70). Only cross-sectional comparisons exist for the inclusion of weight data due to the limitations of spontaneous reporting in the FAERS database, and this study is limited by the current analysis of weight as a single factor. Future studies or clinical trials incorporating body mass index (BMI) or body roundness index (BRI), and using longitudinal change effects, will provide stronger evidence for precise clinical management.

Additionally, increased cumulative AE counts and earlier median onset time of AEs observed with nivolumab correlate with heightened mortality risk. Based on a previous retrospective investigation of PD-1 inhibitor treatment in advanced EC, no irAEs were independent factors for worse Progression-Free Survival (PFS) (71). However, there was no effective grouping evaluation for patients with multiple IRaes. Multiple adverse reactions such as fulminant hepatitis with acute liver failure, immune nephritis with acute renal failure, or triple M syndrome caused by the combination of myocarditis, myositis, and myasthenia gravis can pose a significant risk to the life of patients. Therefore, prospective studies with large sample size and more detailed subgroup analysis are needed to reveal the impact of single or multiple aes on survival outcomes. All in all, these findings collectively underscore the need for precise management and control of irAEs during immunotherapy in esophageal cancer patients. Proactive prevention or timely intervention is essential to enhance patient quality of life and long-term prognosis while optimizing the clinical efficacy of immunotherapy.

### 4.4 Research limitations

However, this study still has several limitations. First, the FAERS database relies on voluntary reporting, with some submissions originating from non-medical professionals, which may introduce reporting bias. Additionally, reports in the FAERS database often lack detailed clinical information, such as disease severity, comorbidities, and medication history, making it more difficult to comprehensively assess drug safety. At the same time, geographical limitations deserve attention, the FAERS database mainly comes from the spontaneous reports of the medical system, patients or pharmaceutical companies in the United States. Due to the differences in medical environment, drug prescription patterns, and patients’ awareness and willingness to report adverse reactions in different countries and regions, the conclusions of this study should be cautiously generalized to other regions of the world (such as Asia, Europe, etc.). Second, FAERS is a spontaneous reporting database without a true exposure denominator and cannot provide incidence rates or valid population-level risk estimates. Furthermore, due to limitations in the number of identified AEs, this study employed only three algorithms, potentially compromising the precision of risk identification. Although disproportionality analysis can be used to assess signal strength, it neither quantifies medication risk nor confirms a causal relationship between a drug and an AE, thus challenging the determination of association. Future studies with larger sample sizes may benefit from combining additional algorithms. Finally, the absence of data from the enrolled population precluded the development of a more robust multivariate risk model. Future multicenter, large-sample clinical data analysis studies exploring the adverse reaction mechanisms of pembrolizumab and nivolumab in esophageal cancer, coupled with the formulation of relevant clinical guidance protocols, will provide more precise safety assessments to support clinical practice.

## 5 Conclusions

Pembrolizumab and nivolumab are the preferred immunotherapies for advanced EC, with combination chemoradiotherapy showing superior long-term outcomes. However, large real-world safety data remain limited. Using the FAERS database, this study firstly analyzed irAEs for these agents in EC patients, identifying high-risk signals such as endocrine, hepatic, renal, and gastrointestinal toxicities, as well as immune-mediated pneumonitis and myocarditis. By subgroup analysis, additional positive signals of cerebral infarction, anxiety/depression, memory impairment, or altered mental status were found in aging patients. Kaplan-Meier analysis with log-rank tests identified earlier occurrence of immune-related AEs in female and aging patients. Univariable logistic regression analysis showed that weight, cumulative number of AE and earlier onset time of AE were associated with higher risk of mortality. These findings may guide personalized treatment and safety management. Additionally, mortality-associated risk factors were identified, which could facilitate early intervention and improve patient prognosis.

## Data Availability

The raw data supporting the conclusions of this article will be made available by the authors, without undue reservation.
